# Mosaic mitochondrial-plastid insertions into the nuclear genome show evidence of both non-homologous end joining and homologous recombination

**DOI:** 10.1186/s12862-018-1279-x

**Published:** 2018-11-03

**Authors:** Shir Portugez, William F. Martin, Einat Hazkani-Covo

**Affiliations:** 1 Department of Natural and Life Sciences, The Open University of Israel, Ra’anana, Israel; 2 School of Molecular Cell Biology & Biotechnology, George S. Wise Faculty of Life Sciences, Tel Aviv University, Tel Aviv, Israel; 3 Institute of Molecular Evolution, Heinrich-Heine University, Düsseldorf, Germany

**Keywords:** Numts, Nupts, Mosaic insertions, Numins, Non-homologous end joining, Gene conversion

## Abstract

**Background:**

Mitochondrial and plastid DNA fragments are continuously transferred into eukaryotic nuclear genomes, giving rise to nuclear copies of mitochondrial DNA (numts) and nuclear copies of plastid DNA (nupts). Numts and nupts are classified as simple if they are composed of a single organelle fragment or as complex if they are composed of multiple fragments. Mosaic insertions are complex insertions composed of fragments of both mitochondrial and plastid DNA. Simple numts and nupts in eukaryotes have been extensively studied, their mechanism of insertion involves non-homologous end joining (NHEJ). Mosaic insertions have been less well-studied and their mechanisms of integration are unknown.

**Results:**

Here we estimated the number of nuclear mosaic insertions (numins) in nine plant genomes. We show that numins compose up to 10% of the total nuclear insertions of organelle DNA in these plant genomes. The NHEJ hallmarks typical for numts and nupts were also identified in mosaic insertions. However, the number of identified insertions that integrated via NHEJ mechanism is underestimated, as NHEJ signatures are conserved only in recent insertions and mutationally eroded in older ones. A few complex insertions show signatures of long homology that cannot be attributed to NHEJ, a novel observation that implicates gene conversion or single strand annealing mechanisms in organelle nuclear insertions.

**Conclusions:**

The common NHEJ signature that was identified here reveals that, in plant cells, mitochondria and plastid fragments in numins must meet during or prior to integration into the nuclear genome**.**

**Electronic supplementary material:**

The online version of this article (10.1186/s12862-018-1279-x) contains supplementary material, which is available to authorized users.

## Background

Mitochondria and chloroplasts are descended from free-living proteobacteria and cyanobacteria respectively. Today’s organelle encodes only a small portion of the genes needed for their function, with 3–60 genes in mitochondria and 23–200 genes in plastids [1]. Despite this genome reduction compared to their free-living ancestors, there are ~ 2,000 proteins functioning within the bioenergetics organelles, the majority of which are encoded on genes in the nuclear genome and whose products are then targeted into the organelles [2, 3]. This reduction in gene number encoded within organelles is explained by a corollary to endosymbiotic theory called endosymbiotic gene transfer (EGT), the transfer of genes from organelle ancestors to the nuclear genome during evolution [4].

In the early phases of organelle evolution, EGT had a massive impact on eukaryote nuclear genome evolution. The process of gene transfer from the endosymbionts to the nucleus had a major role in the origin of eukaryotic genes (Ku et al., 2015) and on the origin of eukaryotic cell complexity [5]. The transfer of organelle DNA to the nucleus is still an ongoing process in most eukaryotes as evidenced by the finding of fragments similar to mitochondria and plastids within the nuclear genome (reviewed in [6]). Insertions originating from mitochondrial DNA are termed numts [7], insertions from plastid DNA are called nupts [1].

Numts and nupts are continuously inserted into the nuclear genome, as is best observed by variation within populations. For example, an analysis of 1000 human genomes uncovered 141 numts that are polymorphic among humans [8], the evolutionarily most recent insertions tracing to the Chernobyl event [9]. The transfer of numts and nupts from organelles to the nucleus has also been shown experimentally, both in plants [10, 11] and in yeast [12, 13].

Available data indicate that numts and nupts are captured into double-strand breaks (DSBs) in the nuclear genome during their repair by the non-homologous end joining mechanism (NHEJ) [13–15], one of the two cell mechanisms for double-strand break repair. In this mechanism, filler DNA may be inserted into the lesion during the repair process, which often entails deletion of short DNA segments surrounding the break. When numts and nupts are used as filler DNA during repair via NHEJ, deletion involves fewer bases relative to NHEJ in which non-organelle DNA serves as filler [16, 17]. Two common, but alternative, hallmarks of NHEJ are observed in numt and nupt insertions: microhomology, a short homology of 1–7 bp between the organelle DNA edge and the nuclear DNA, and a blunt-end repair that occurs without homology [14, 16].

Complex insertions of numts and nupts are defined as multiple stretches of organelle DNA in nuclear chromosomes that originated from different locations and orientations of the organelle genome. Such complex events have been reported both for numts [13, 14] and for nupts [18, 19]. Junctions between organelle fragments in such events can show typical NHEJ signatures. Examples of complex events include nupt insertion with different fragments and polarity in rice, *Arabidopsis* and tobacco [18, 19]. In addition, complex insertions were also identified for numts in yeast and humans [13, 14].

It was initially unclear whether these insertions rearranged prior, during or after the integration into the nuclear genome. Events that were found to span a long nuclear distance were reported as occurring after integration as part of the nuclear chromosome evolution [19–23]. However, experimental EGT studies showed complex rearranged numts as well as complex rearranged nupts with NHEJ signatures, suggesting that complex events are formed before or during the integration into the nuclear DNA [11, 24–29]. A recent analysis of numts and nupts in 44 fully sequenced genomes [30] showed that the number of complex insertions varies dramatically among species.

Nuclear mosaic insertions (numins) are a special case of complex insertions in that they involve the insertion of both mitochondrial and plastid at the same nuclear location. The prevalence of numins, as well as their mechanism of formation, is unclear, as these unique insertions have rarely been analyzed. Early studies by Richly and Leister [31, 32] showed a small number of organelle fragments that are up to 5 kb apart: *Arabidopsis* harbours 10 such insertions while rice chromosomes 1, 4 and 10 harbour 33. Noutsos et al. [22] reported five numins that vary in size between 3-92Kb in *Arabidopsis* and rice. These numins were reported as being composed of up to 100 different segments originating from the two organelles, but signatures of NHEJ or of homologous recombination were not detected and the mechanism of integration remained unclear. Fragments that are nearby but not adjoining can be the result of later events occurring as part of the evolutionary dynamics of the nuclear genome [33]. Wang and Timmis [17] identified 14 recent organelle insertions that are unique to *Oryza sativa subsp indica* but absent from *O. sativa subsp*. Japonica, with three of the recent insertions are of mosaic origin bearing the familiar NHEJ signatures known from numts and nupts. Only adjacent fragments can reveal signatures reflecting the mechanisms during integration into the nuclear genome. Here we quantify and characterize numins in nine plants and elucidate their insertion mechanism.

## Results

### Numins contribute up to 10% of the total organelle insertions

To estimate the prevalence of nuclear mosaic insertions (numins), BLAST was first used to search the nuclear genomes of nine plants with their corresponding mitochondrial and plastid genomes. We defined the term fragment to describe a single nuclear locus with homology to organelle DNA (see Methods). The number of independent organelle insertions was inferred using concatenation of fragments (see Methods) [30]. In total, five classes of inferred insertions were identified: (a) simple numts (a single mitochondrial fragment); (b) simple nupts (a single plastid fragment); (c) complex numts (multiple mitochondrial fragments); (d) complex nupts (multiple plastid fragments); and (e) numins (both mitochondrial and plastid fragments).

**Fig. 1:**
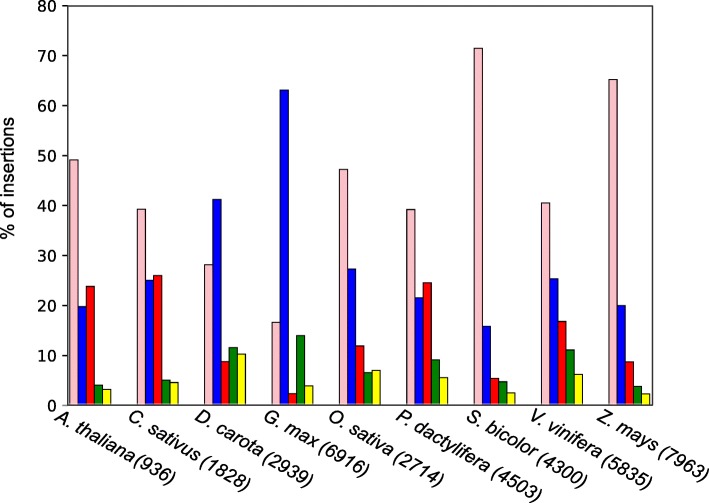
Distribution of organelle insertions in the genome of nine plant species. Simple numts composed of a single mitochondrial fragment (pink), simple nupts composed of a single plastid fragment (blue), complex numts composed of a number of mitochondrial fragments (red), complex nupts composed of a number of plastid fragments (green) and numins composed of at least one mitochondrial and one plastid fragment (yellow). The total number of inferred insertions is indicated for each species

Figure 1 shows the frequency of the five classes of organelle insertions with a concatenation distance of 5 kb in the analyzed nine plant genomes (listed in Additional file 1: Table S1). In total, we identified 1,782 numins in all genomes tested. The frequency of numins relative to all organelle insertions in each organism ranged from 2% (183 out of 7,963) in *Z. mays* to 10% (302 out of 2,939) in *D. carota*. Of note, changing of the permitted concatenating distances has no effect on the trend of results, as previously shown for single origin insertions [30]. In the following, we chose to present results of inferred insertions with a distance of up to 5 kb.

Simple numts and nupts are the most frequent organelle insertions in all studied organisms, with *numts* dominating the genomes of *A. thaliana, C. sativus, O. sativa, S. bicolor, V. vinifera, Z. mays* and *P. dactylifera,* and nupts dominating the genomes of *D. carota* and *G. max*. The single organelle complex insertions are substantially more frequent than numins in all organisms. In addition, when numts are more frequent than nupts, complex numts are the most common type of complex insertions, and vice versa. Thus, simple nupts dominate in *G. max* with 63% of the total organelle insertions, while complex nupts dominate the *G. max* with 14% of the complex organelle insertions. Similarly, simple numts dominate *P. dactylifera* with 39% of the total organelle insertions while complex numts dominate the *P. dactylifera* with 25% of the complex organelle insertions. We also tested if the meeting of organelle fragments is random, that is, if a junction between two fragments occurs with comparable frequency independent of their organelle origin. Simulation of the organelle origin of all fragments shows that mitochondrion and plastid fragments do not meet randomly; pairs of fragments from the same origin (mitochondria-mitochondria or plastid-plastid) are overrepresented while mosaic pairs are underrepresented in real data (*p* value < 0.05).

### Numins show NHEJ signatures

Signatures reflecting the mechanism of integration into the nuclear genome are of interest. Numts and nupts are known to integrate into nuclear DSBs via NHEJ. Two hallmarks of NHEJ are known: microhomology and blunt-end repair. We compared the signatures of complex insertions originating from complex numts and complex nupts to numins. Because NHEJ signatures can only be detected by looking at nearby fragments of inferred insertions, only inferred insertions with adjacent mitochondria and plastid fragments were further analyzed. To estimate the length of microhomology, each of the corresponding organelle fragments was extended by 10 bp and the number of overlapping bases until the appearance of a mismatch was counted. In our dataset of inferred insertions with a distance of up to 5 kb, there are 3,206 dual origin junctions. Of these junctions, 1,503 are up to 10 bp apart and were considered for the NHEJ analysis.

NHEJ signatures of complex numts and nupts were reported both between adjacent organelle fragments (inner junctions) and between the terminal organelle fragments and the nuclear genome [13, 14]. Analysis of the terminal junctions requires the availability of highly similar nuclear genomes [16, 17]. Since the similarity between the available plant genomes is not high enough, our analysis only considered the inner junctions while terminal junctions were not considered.

**Fig. 2:**
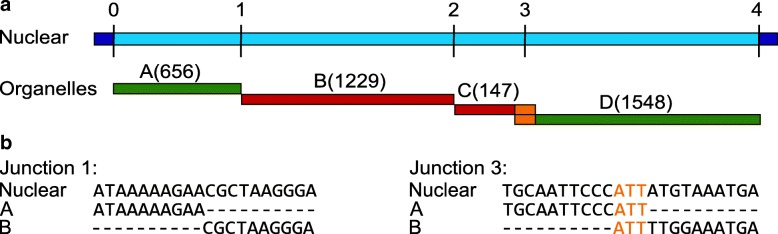
An inferred numin in the genome of *Z. mays* shows NHEJ signatures. **a** The total length of this inferred insertion located on chr5:210,342,490-210,346,062 is 3,573 bp (light blue) and it is composed of four fragments: two originating from the mitochondria (red) and two originating from the plastid (green). The junction number is indicated above the nuclear DNA and the length of each DNA fragment is indicated. Note that only junctions between organelle fragments are analyzed (1–3) while the terminal junctions with the nuclear genome (0,4) are not analyzed. **b** Two junctions between mitochondria and plastid fragments are shown at the base pair level. Junction 1 between fragments A (plastid) and B (mitochondrial) shows blunt-end signature while junction 3 between fragments C (mitochondrial) and D (plastid) shows microhomology of three bases (ATT, in orange)

Figure 2 demonstrates an example of numin in the genome of *Z. mays*. Two plastid fragments and two mitochondrial fragments form an insertion of 3,573 bp. Detailed examples of two inner junctions are shown in Fig. 2b. Junction 1 between plastid fragment A and mitochondrial fragment B shows blunt-end repair. In contrast, junction 3 between mitochondrial fragment C and plastid fragment D shows three bases of microhomology. In this case, the bases ATT that appear in the nuclear genome are shared between the mitochondrial and the plastid genomes.

**Fig. 3:**
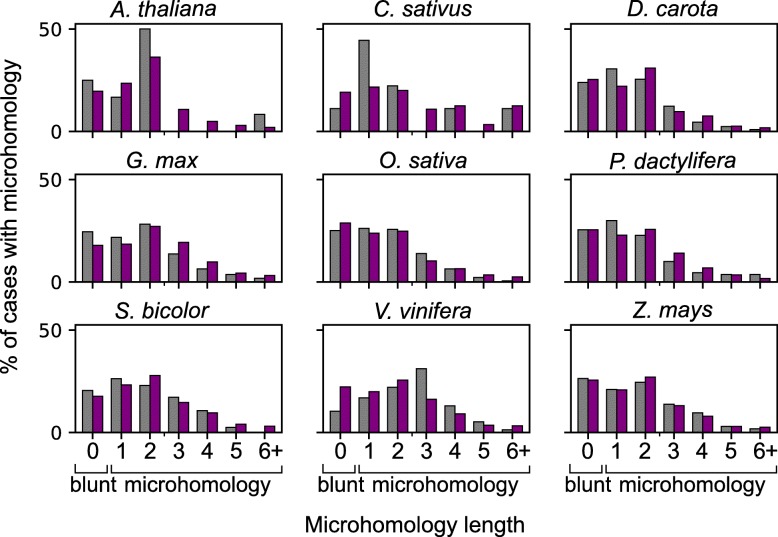
Microhomology length distribution for internal junctions of complex insertions in nine plants. Single origin insertions are shown in purple and mosaic origin insertions are shown in gray. Blunt end is shown as microhomology of length 0

Comparison of microhomology length between junctions that are of dual origin and junctions from single organelle origin are shown in Fig. 3. The distribution of the number of microhomology bases is similar in the two sets and both blunt-end repair and microhomology were identified. Statistical analysis using multinomial tests shows that we could not reject the null hypothesis that junctions from dual origin have similar microhomology distribution to those of single origin (*p* value > 0.05). However, in *D. carota* and *V. vinifera* the test suggests that the distributions are different. The *V. vinifera* seems to have more blunt-end junctions in the single organelle insertions while dual organelle junctions seem to have more of 3 bp microhomology junctions. Our results suggest that NHEJ is a key mechanism in numins and that the mosaic insertion mechanism is similar to that of single-origin complex insertions.

While only a handful of numins events with NHEJ signatures were previously observed [17], our data suggest that NHEJ is a major mechanism in numins. Our results indicate that 1,128 out of 3,206 (35%) junctions that appear between mitochondria and plastid fragments show microhomology or blunt-end signatures.

### NHEJ signatures do not reflect recurring insertion events

Two circumstances can explain the presence of NHEJ signatures between two organelle fragments either from a single origin or from a dual origin. (a) In the case of single insertions, complex numts and nupts are concatenated before or during the capture into DSBs (this is the current consensus). (b) In the case of recurrent insertions, DSB hotspots might exist in these loci [22] and complex insertions could result from multiple insertions into the same locus in the nuclear genome. That is, first an organelle fragment is captured in a DSB in the nuclear genome and later the nuclear genome undergoes a second DSB at the same locus where a second fragment is captured. If the latter mechanism is frequent, numins would provide an opportunity to detect it.

We looked for evidence of recurring events by analyzing numins in *O. sativa* subsp*. japonica* and comparing them to insertion events in *O. sativa* subsp*. indica* [17], but found no cases that would reflect a recurrent insertion mechanism. This suggests that the same mechanism operating for numts and nupts is also responsible for the integration of numins. Thus, it appears that the concatenation of mitochondria and plastid fragments in numins occurs before or during the integration into the DSB in the nuclear genome, requiring the coexistence of free DNA from both organelles somewhere in the cell, possibly nucleoplasm or autophagosomes, prior to insertion.

### Complex insertions show long homology

Complex insertions can potentially be the result of homologous recombination or single-strand annealing (SSA) between fragments either during or after integration. Such long homology could not be identified in previous studies of primates [16]. However, in the present sample of plant genomes, we identified cases with long homology by screening insertions for organelle fragments whose sequence overlapped by at least 40 bases, a homology stretch that is too long for NHEJ. A fragment that was degraded in the nuclear genome can mistakenly be identified as two overlapped organelle fragments with long homology. To prevent false identification of insertions that occurred through long homology mechanisms, we set a criterion such that the overlapping fragments cover at least 100 base pairs that are not shared with other fragments.

**Fig. 4:**
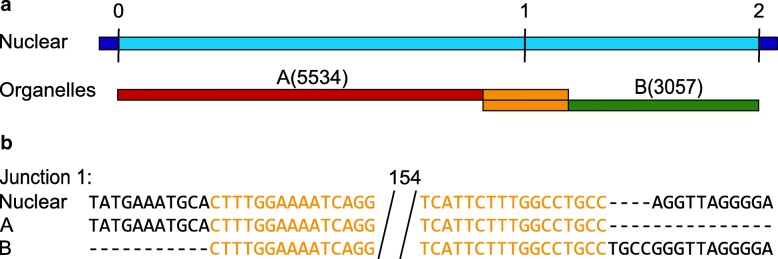
An inferred numin in the genome of *Z. mays* shows long homology. **a** A complex insertion located in chr4:156,747,273-156,755,671 is composed of two fragments that are 5,534 bp and 3,057 bp overlapping by 186 bp. **b** The junction between mitochondria and plastid fragments is shown at the base pair level. Overlapping between mitochondrial and plastid fragment is of 186 bp and identity in the overlapping region is 100%. The overlapping region is trimmed and the size is indicated

We identified 16 such events (Additional file 2: Table S2) in complex numts, complex nupts, and numins. An example of a complex numt in *Z. mays* chr4:156,747,273-156,755,671 is shown in Fig. 4. This numt is composed of two mitochondrial fragments that are 5,534 bp and 3,057 bp long, overlapping by 186 bp.

In addition to these 16 events that are unique in each genome, the grape nuclear genome shows insertions with long homology that appear multiple times. The most extreme example is an insertion of four mitochondrial fragments with overlapped fragments of up to 440 bp appearing at least 38 times in the nuclear genome. These copies are highly similar to each other and to the mitochondrial genome. It is unclear how these insertions composed of the same fragments evolved multiple times in the genome. It seems unlikely that the same insertion integrated independently multiple times. Interestingly, one of the four fragments of this insertion includes mitochondrial *orf333* which encodes a reverse transcriptase LTR, suggesting that these might be duplicated copies of one or a few insertions.

### NHEJ signatures are enriched in recent numins

Our finding of NHEJ in 35% of numin junctions with adjacent fragments might be an underestimation of that mechanism, because numts and nupts are degraded by mutations after integration as part of nuclear genome evolution [19–22]. This process can give rise to longer distances between insertion fragments [32] damaging NHEJ signatures. Therefore, numins with NHEJ signatures should show a higher similarity to organelle DNA than numins without NHEJ insertions.

To test that, we labeled insertions as NHEJ or non-NHEJ. NHEJ insertions were defined as those that contain at least one junction with zero to ten base pair overlap between fragments. Similarly, we labeled insertions as non-NHEJ if all of their fragments are separated by at least one base pair. Insertions with long homology between fragments were omitted to avoid a contamination by SSA or gene conversion mechanism.

**Fig. 5:**
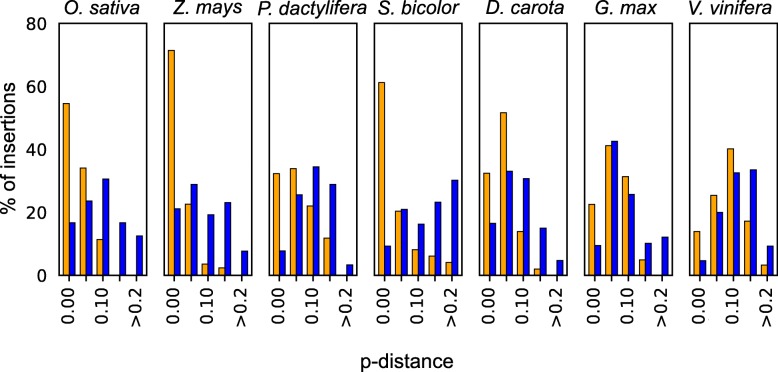
Distribution of p-distance in NHEJ (orange) and non-NHEJ (blue) mosaic insertions in seven species. NHEJ insertions are defined as insertions with at least one junction with 0–10 base pairs overlap between fragments. Non-NHEJ is defined as insertions where all of their fragments are spaced by at least 1 base pair

We calculated a p-distance for each numin for a total of 1563 insertions. This number includes 750 NHEJ insertions and 813 non-NHEJ insertions. The p-distance distribution for seven organisms with at least 100 numins (Fig. 5) shows that NHEJ signatures are enriched in recent numins. Indeed, Mann Whitney test shows that NHEJ p-distance is significantly lower than non-NHEJ p-distance for all organisms analyzed (one-tail *p* value < 10^−5^). Thus, the number of identified NHEJ insertions in our data is probably an underestimate of insertions integrated via this mechanism.

## Discussion

### Numins show NHEJ signatures

The present work is an attempt to systematically identify nuclear mosaic insertions (numins) in plant genomes and to infer the mechanism of their integration. We focused on adjacent fragments in nuclear genomes in order to assess their NHEJ signatures and to compare them to those of complex numts and numts. Comparing the NHEJ junction signatures of numins (fragments from two different organelles) to those of single origin (two fragments from one organelle) revealed no marked differences relative to single origin insertions. The results indicate that 35% of the junctions reveal NHEJ signatures, suggesting that NHEJ is a key mechanism in numins integration. Our findings that NHEJ signatures are enriched in recent numins suggest that they have been mutationally eroded in older ones, such that identified cases NHEJ in numins are systematically underestimated.

Previous findings regarding complex insertions from a single organelle show that they can include fragments from various regions of the organelle DNA, sometimes in opposite orientations [30]. These complex insertions are formed before or during integration into nuclear DSBs [22]. Single and dual origin insertions show similar NHEJ signatures. It is thus likely that, similar to complex insertions from a single origin, numins undergo concatenation before or during integration into the DSBs. Though we found no evidence of hotspots for numins, the present sample is limited.

### Complex insertions can be the result of gene conversion

The results indicate that complex insertions can undergo gene conversion or SSA. To our knowledge, evidence for mechanisms involving long homology in organelle insertions was hitherto lacking. Gene conversion occurs when a previously inserted fragment is partly replaced by a new fragment leaving two traces: a leftover of the first fragment and long homology between the two fragments. A similar outcome can be the result of SSA, a DSB repair pathway that involves the annealing of homologous repeat sequences in the flanking side of the DSB. SSA involves end-resection and thus deletions occur during the repair process similar to the case of NHEJ [34]. However, while gene conversion can occur even without a DSB, integration of additional fragments into the nuclear genome via SSA requires the formation of a new DSB. Thus, long homology can be the result of two types of events: one that occurs before or during integration and one that occurs after integration. It is not surprising that evidence for long homology was identified in plants as opposed to primates, since plant organelles are enriched with repeats both within organelle [35] and between the two organelles [36, 37].

The observation that long homology can be involved in the integration of organelle DNA suggests that other mechanisms in addition to NHEJ participate in organelle integration. The effect of NHEJ depletion on the transfer rate of numts, nupts and numins can be tested with organelle transformation systems that activate selectable markers in the case of organelle transfer into the nuclear genome, such as the Thorsness and Fox system [12] for numt selection in yeast and the Timmis system for nupts selection in tobacco [11, 24].

### When and where do mitochondria and plastid meet?

The formation of numins before or during integration into the nuclear genome requires an additional step - the meeting of mitochondrial and plastid fragments. This step is not required for the insertion of numts or nupts as they have a shared history in the mitochondria or in the plastid. Compared to simulated data, pairs of fragments of the same origin are overrepresented in real data, while pairs of mosaic fragments are underrepresented. Where and how DNA from different organelles comes into contact is unknown.

It was previously suggested that numins could have occurred under stress conditions that affected both organelles [3]. In flowering plants, organelles are maternally inherited so transfer can occur during organelle degradation in the course of male gametogenesis [1]. The transfer of DNA fragments from one organelle to another (mitochondria to plastid or vice versa) followed by relocation of the chimeric DNA fragment to the nucleus is an option. However, as reported in the literature, the number of such transfer events between the organelles is relatively low. These cases include the transfer of plastid DNA into the mitochondria [37–39] as well as transfer from the mitochondria to the plastid [36, 40, 41].

Using selective autophagy, plants can remove mitochondria and chloroplasts in vacuolar processes called mitophagy and chlorophagy respectively [42]. Previous studies in yeasts showed that mutations in a few nuclear genes, called the *yme* (yeast mitochondrial escape), increase mitochondrial escape to the nuclear genome [12, 43]. At least one of these *yme* mutants causes elevated escape of mitochondrial DNA by targeting abnormal mitochondria for degradation by the vacuole through mitophagy [44]*.*

A recent paper by Diner et al. [45] suggested a surprising meeting place in episomes within the nucleus of diatom. These unicellular photosynthetic eukaryotes can maintain circular artificial chromosomes of foreign DNA sequences in the form of episomes. These episomes were suggested to be composed of numts and nupts, among other foreign DNA sequences, and can recruit centromeric proteins. All these locations are potential places for mitochondria and plastid DNA encounters prior to integration.

## Conclusions

Numts and nupts have been extensively studied in eukaryotic genomes and underscore the ongoing nature of EGT. However, little is yet known about numins. The common NHEJ signature that was identified here reveals that, in plant cells, mitochondria and plastid fragments must meet during or prior to integration into the nuclear genome. Where in the cell the DNA that comprises mosaic insertions combines remains unknown, but autophagy might be involved.

## Methods

### Data

Nine plant species with their mitochondrial, plastid and nuclear genomes were downloaded from NCBI. Genomes include: *Arabidopsis thaliana* (NC_003074.8, NC_003076.8, NC_003071.7, NC_003075.7, NC_003070.9, NC_001284.2, NC_000932.), *O. sativa* subsp. japonica (NC_008395.2 - NC_008405.2,NC_008394.4, NC_011033.1, NC_001320.1), *Vitis vinifera* (NC_012007.3 - NC_012025.3, NC_012119.1, NC_007957.1), *Sorghum bicolor* (NC_012870.1 - NC_012879.1, NC_008360.1, NC_008602.1), *Glycine max* (NC_016088.2 - NC_016107.2, NC_020455.1, NC_007942.1), *Zea mays* (NC_024459.1 - NC_024468.1, NC_007982.1, NC_001666.2), *Cucumis sativus* (NW_011953803.1 - NW_011953809.1, NC_016004.1 - NC_016006.1), *Daucus carota* (NW_016089416.1 - NW_016089424.1, NC_017855.1, NC_008325.1), and *Phoenix dactylifera* (NW_008246507.1 **-** NW_008326821.1,NC_016740.1, NC_013991.2).

### Identification of organelle DNA in nuclear genomes

The mitochondrial and plastid genomes of each plant species were BLASTed against the corresponding nuclear genome using BLASTN (BLAST+, [46]). The following parameters were used: dust was set to yes and the E-value was set to 0.001. Blast hits that were nested within other BLAST hits were eliminated.

### Inferring the number of numts, nupts and numins

We defined the term fragment to describe a blast hit that maps a region in the nuclear genome to a region in either the mitochondrial or plastid genome. Of note, because the mitochondria and plastids harbor repetitive genomic regions, a single fragment can map to more than a single mitochondrial or plastid location. In some cases the overlap in the nuclear location between two blast hits was so high that we joined them and considered them as a single nuclear fragment. Specifically, if one blast hit overlapped the other blast hit in all but a few bp they were united. Here, fragments were united if this non-overlap was ≤10 bp.

We denote by *n* the number of fragments that characterize each inferred insertion. In order to infer insertions, fragments were concatenated based on a maximal distance between the nuclear coordinates without any consideration of organelle coordinates or orientation. The permissive concatenation described for numts and nupts [30] was applied here on fragments from both mitochondrial and plastid origin. The following distances were considered: 3 kb, 5 kb, and 10 kb. The estimated number of insertions is inferred from the described concatenations.

Inferred insertions can be either simple, if they are composed of one fragment, or complex if they are composed of multiple fragments. Simple insertions can be either numts or nupts. Complex insertions, on the other hand, can be mitochondrial (numts) if all of their fragments are of mitochondrial origin, plastid (nupts), if all their fragments are of plastid origin, or numins. A complex insertion is classified as numin only if it has at least one certain mitochondrial fragment and one certain plastid fragment. Inferred insertions can, however, include fragments that are of ambiguous origin if they are composed of BLAST hits of both mitochondrial and plastid origin (due to repetitive sequences shared between the organelles themselves).

In order to test if the occurrence of juxtaposed mitochondrial and plastid fragments is random, we counted the number of junctions in complex insertions. The term junction describes the joining between two adjacent fragments of complex insertions (inner junctions) or the joining between the insertion’s external fragments and the nuclear genome (terminal junctions). In our analysis, only inner junctions are considered. The term junction is therefore used to describe inner junction. A junction can be classified as one of three types: mitochondrion-mitochondrion, plastid-plastid, and plastid-mitochondrion (mosaic). We then compared the number of junctions of each type to the number of predicted junctions based on a simulation. The simulation was done using a permutation test. For each organism, we shuffled the organelle origin of all fragments, while retaining the original nuclear location. This procedure was done 10,000 times, to generate a null dataset of organelle insertions. The number of junctions of each type was counted for each permutation and the means of these counts were used as expected values. We scored each permutation with an appropriate Cressie-Read power divergence statistic to generate a sampling distribution of this test statistic. We then tested whether the meeting of mitochondrion and plastid fragments is different from the null distribution.

### NHEJ analysis

The known integration mechanism for numts and nupts is NHEJ [13]. This mechanism is characterized by two types of signatures: microhomology and blunt-end repair. These signatures can only be identified between adjacent fragments. Therefore for this analysis, only complex insertions with fragments that are 0–10 bp apart were considered. Only unambiguous junctions that are classified as mitochondrion-mitochondrion, plastid-plastid or plastid-mitochondrion were used. A junction is classified as single-origin if it is composed of fragments that are mitochondrion-mitochondrion or plastid-plastid. A junction is classified as dual-origin if it is composed of fragments from both organelles (mitochondrion-plastid or plastid-mitochondrion). Of note, junctions in numins can also be single-origin (mitochondrion-mitochondrion, plastid-plastid) but these were excluded from the analysis.

For each junction, we looked for the appearance of microhomology, identified as overlapped bases between the nuclear fragments. For that, each of the corresponding organelle fragments was extended by 10 bp and the number of overlapped bases until the appearance of mismatch was counted. For example, if the end of the first fragment in the concatenation is AATT**TTG** and the beginning of the second fragment in the concatenation is **TTG**AAAC then the overlap is TTG and the microhomology is of length 3. If no microhomology was identified (microhomology of length 0) the junction was classified as blunt-end.

In order to test whether junctions that are of dual origin show the same NHEJ signatures as junctions of single origin, we performed a multinomial test. Specifically, we tested if microhomology length distribution observed in dual-origin junctions comes from the same distribution observed in single-origin junctions. Due to small sample size, microhomology of 6 bp or more was binned into the same category, to form a total of *k* = 7 categories (*i* = 0,…,6). The set of junctions that are of single-origin forms the null dataset and the dual-origin junctions are the observed data.

### Comparative genomics of rice

To test the mechanism of integration of numins, we looked for evidence for recurring integration events. Nuclear genomes of two closely related species, *O. sativa* subsp. *japonica* and *O. sativa* subsp*. Indica,* were chosen for that analysis. Each of the numins identified in *O. sativa* subsp. *japonica* was extracted with extension of 500 bp to each side. Using BLASTN we searched for the existence of each insertion in the nuclear genome of *O. sativa* subsp. *indica*. Specifically, cases where a full fragment is missing in *O. sativa* subsp*. indica* but the flanking regions of the insertion can still be identified in close proximity in *O. sativa* subsp*. japonica* were considered. *O. sativa* subsp*. Indica* accessions are CM000126-CM000137.

### Insertions with long homology

Complex insertions composed of organelle fragments that overlap by at least 40 bp by BLASTN and whose overlapping fragments have at least 100 additional non-overlapping bp, were considered probable recombination events. These were further tested manually to exclude non-reliable examples.

### Calculating the p-distance of numins

To test whether insertions with NHEJ hallmarks are enriched in recent insertions we calculated the p-distance of each numin from its corresponding organelle. The p-distance calculation was done using the following formula:

$$ \mathrm{p}\hbox{-} \mathrm{distance}={\sum}_{i=1}^n\frac{mismatches(i)+ gaps(i)}{len(i)} $$

Insertions with long homology (above 10 bp) were excluded from analysis, since they are suspected to be integrated into the nuclear genome via a different mechanism. One-tailed Mann-Whitney test was used to test if the p-distance of numins with NHEJ is less than that without NHEJ.

## Additional files

Additional file 1: **Table S1.** Organelle insertions identified in nine plant species. Table contains information about the location of all events (numts, nupts, and numins) identified in the nine species analyzed. (XLSX 1714 kb)

Additional file 2: **Table S2.** Complex insertions with long homology. Table contains information about 16 insertions that show long homology. (XLSX 13 kb)

## Abbreviations

numins: Nuclear mosaic insertions
numts: Nuclear copies of mitochondrial DNA
nupts: Nuclear copies of plastid DNA
